# A model of puppy growth during the first three weeks

**DOI:** 10.1002/vms3.322

**Published:** 2020-07-03

**Authors:** Isabel Alves

**Affiliations:** ^1^ Instituto Superior de Agronomia Universidade de Lisboa Tapada da Ajuda LISBOA Portugal

**Keywords:** growth chart, growth rate, neonatal period

## Abstract

Neonatal mortality in puppies is highly variable, with large scale surveys still reporting average values around 10% –15%. Weight measurement is the simplest way to monitor the development of the puppies, and a weight loss during the first 48 hr has been recognized as one of the factors that puts puppies at a higher risk of neonatal mortality. However, little is known about what constitutes optimum growth up to 3 weeks. In this study, a mathematical formula with the form *P = P*_0_ exp (0.13084 *x* ‐ 0.001616 *x^2^*), where *P* is weight on Day *x* and *P*_0_ is weight on Day 0, obtained by multiple linear regression, is presented and validated with data from 345 puppies belonging to 60 litters of 19 different breeds, from toy to giant size, showing that it appropriately describes maximum puppy growth rate during the neonatal period for all breeds. This formula is in agreement with previous studies and generic recommendations that can be found in the literature on puppy growth from birth to 21 days regarding relative daily weight gain. It can be easily introduced in a spreadsheet or used to build growth charts that can help the breeder or the veterinarian in monitoring and evaluating puppy growth during the neonatal period. Although deviations from the maximum growth rate can now be quantified, there is still a need to determine the limits beyond which supplementary feeding is advised/required.

## Graphical abstract

Maximum puppy weight growth rate during the first 3 weeks can be described by an equation of the exponential type.Puppy weight can be predicted with this equation given weight on Day zero (birth day) and age of the puppy This equation is shown to properly describe puppy weight increase, irrespective of birth weight and breed

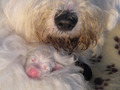

## 1 INTRODUCTION

The neonatal mortality in puppies (from birth to weaning) is highly variable, with large‐scale studies still reporting average values around 10%–15% (Chastant‐Maillard et al., 2017; Tønnessen, Sverdrup, Borge, Nodtvelt, & Indrebo, 2012) but increasing to 20%–30% in some breeds or kennels (Soares, Dourado, Alves, & Mateus, 2019; Tønnessen et al., 2012; Vassalo et al., 2015). Several factors have been implicated, such as quality and duration of labour, congenital/hereditary anomalies, maternal ability of the bitch, environmental conditions, nutrition or infectious diseases (Carmichael, 2004; Davidson, 2003), with the majority (75%–90%) of losses occurring during the first 3 weeks (Indrebø, Trangerud, & Moe, 2007; Mila, Grellet, Feugier, & Chastant‐Maillard, 2015), when puppies are dependent exclusively on milk consumption.

The simplest and most inexpensive way to monitor puppies and recognize problems early is to weigh them regularly (Lawler, 2008; Wilsman & van Sickle, 1973), preferably daily for the first two weeks, then at least every 3 days until one month of age (Kirk, 2001). Mila et al. (2015) showed that variation in weight during the first 48 hr was linked to the mortality between 2 and 21 days of age, with a birth weight loss of 4% or more leading to a higher risk of mortality. Wilsman and van Sickle (1973) also reported a lower mortality in puppies gaining weight since immediately after birth or losing less than 10% of their birth weight during the first 48 hr. Despite the interest of these generic limits to monitor early growth and identifying puppies at risk, a more detailed description of weight gain of puppies during the first three weeks, when they are exclusively milk fed, is needed. However, this kind of information is still scarce and often relative to a specific breed (Bigliardi, Di Ianni, Parmigiani, Morini, & Bresciani, 2013; Schroeder & Smith, 1994), with most studies being made on the period from weaning to adulthood (Dobenecker, Endres, & Kienzle, 2013; Salt et al., 2017).

The objective of this work was to develop a model that appropriately describes weight gain in puppies from birth to 3 weeks that can be applied to several breeds, regardless of size and conformation. Such a model can help identifying the puppies that are growing below what is to be expected but also to quantify that deviation, which can help the breeder/veterinarian to define the need for supplementary feeding.

## 2 MATERIAL AND METHODS

### 2.1 Model development

**Figure 1:**
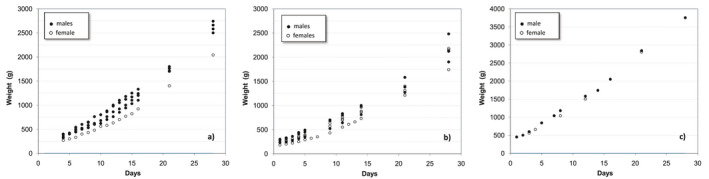
Evolution of the weights of the puppies up to 28 days after birth. (a) litter A; (b) litter B; (c) litter C

The model was first developed using data from three litters of Old English Sheepdogs (OES) from the same breeder, as a tool to describe and evaluate the development of puppies in this kennel during the first weeks after birth. The first litter (litter A) consisted of five live puppies (four males and one female), with another 6 being stillborn or dying during the first 48 hr. Weight monitoring was daily performed, at about the same time of the day, from Day 4 to Day 21 using the same scale (Stube, model 205, Longare, Italy), with 5 g divisions and a maximum charge of 4 kg. Despite protecting the smaller female from the competition with her littermates during nursing time, the difference in weight to the males increased progressively (Figure 1a). Nevertheless there were no other apparent differences between the puppies, namely regarding activity or development.

In the second litter (litter B), that consisted of three males and four females (plus three stillborns), the evolution was similar to that observed in the first litter, and again showing a progressive divergence between the weight of the bigger and the smaller puppies (Figure 1b).

The third litter (litter C) consisted of only two puppies (one male and one female), of about the same size and heavier than the puppies from the previous litters, that evolved similarly (Figure 1c).

None of the puppies received additional feeding and were exclusively fed by their dams.

**Figure 2:**
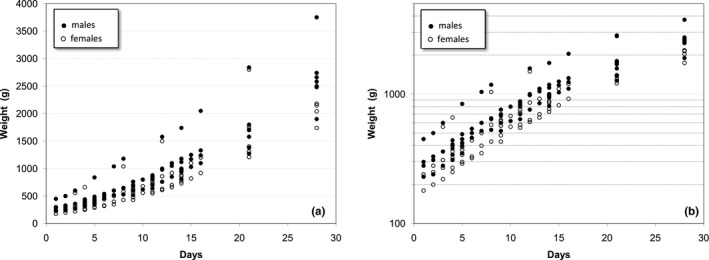
Evolution of the weights of the puppies up to 28 days after birth (all litters). (a) normal scale; (b) logarithmic scale

Figure 2a, with all the data plotted together, suggests that a common model cannot be applied to all puppies as the different curves have different slopes. However, when the data are plotted in logarithmic scale (Figure 2b), the curves representing the weight increase of the puppies are parallel, denoting an identical rate of weight gain.

The modelling then consisted of finding the common slope coefficients of the different individual curves, differing only in the intercept with the *y* axis. Besides the age in days (*x* variable) and the measured weight (in g) (*y* variable), 14 ( = number of puppies) new variables were added, *I_1_*, *I_2_*, ⋯, *I_14_*, that take the value 1 or 0 depending on the weight value referring to that puppy or not. A measurement of puppy number 1 thus received the values *I_1_* = 1, *I_2_*, ⋯, *I_14_* = 0; a measurement from puppy number 2 was identified as *I_1_* = 0, *I_2_* = 1, *I_3_*, ⋯, *I*_14_ = 0; and a measurement from the last puppy was recorded as *I_1_*, ⋯, *I_13_* = 0, *I_14_* = 1. The GENSTAT statistical package was used to adjust a polynomial to the logarithmized *y* values, with the form:

$\ln\;y=\sum_{i=1}^{14}a_{i}I_{i}+\sum_{j=1}^{n}b_{j}x^{j}$

The coefficients *a*_1_, *a*_2_, ⋯, *a*_14_ therefore correspond to the weight at Day 0 (birth day) (*P*_0_) of the different puppies (*a_i_* = ln *P*_0_ *_i_*), and *b*_1_, ⋯, *b_n_* are the (common) regression coefficients. Data from all puppies were combined together, regardless of sex, as average daily growth rate of males (11.5% ± 5.3%) was found to be not statistically different from the correspondent average daily growth rate of females (11.6% ± 4.4%) (*p* =0 .92, *t* Student test). Several polynomials, with increasing order *n* (up to *n = *4), were tested and the values of R^2^/R^2^_adj and the confidence interval (at the 95% level) of the regression coefficients analysed.

### 2.2 Model validation

The model was first validated with other litters of OES, both from the same breeder and another four litters from two other kennels working with unrelated lines (no common dogs in at least the previous three generations), in a total of 12 litters and 75 puppies (33 males and 42 females). Birth weight of these puppies ranged from 265 g to 510 g.

The curves were also tested on litters from other breeds, first on breeds of similar morphological type and adult weight (Belgian Shepherd) and then on both smaller and larger breeds of different types of conformation (including molossoids, sighthounds and achondroplasic breeds), with birth weights ranging from 70 to 1,000 g (Table 1). All puppy weights were recorded by the respective breeders. Besides converting dates in days after birth, no other transformation/cleaning was made. All the puppies were alive at the end of the period of interest (21 days).

**Table 1:** Data relative to the litters and breeds used for validation

Breed	Adult weight, kg	Number of litters	Number of puppies/litter	Total number of puppies (♂/ ♀)	Puppy weight at birth, g
Chihuahua	1.5–3	4	2–4	12 (5/ 7)	130–145
Yorkshire Terrier	up to 3.2	2	4–5	9 (5/ 4)	70–165
Portuguese Podengo	4–6	2	4–5	9 (4/ 5)	115–200
Lhasa Apso	6–9	1	2	2 (0/ 2)	130–150
Parson Russel Terrier	6.5–8.5	2	4–6	10 (6/ 4)	100–250
Cavalier King Charles Spaniel	5.4–8	5	2–7	25 (15/ 10)	130–230
Shetland Sheepdog	7.5–12.5	1	4	4 (2/ 2)	200–280
Standard Dachshund	< 9	1	6	6 (4/ 2)	240–340
Whippet	12.5–20	5	6–9	39 (22/ 17)	240–400
Bulldog	23–25	3	4–6	16 (7/ 9)	240–400
Bull Terrier	25–35	2	1–7	8 (4/ 4)	300–450
Shar Pei	22.5–30	4	3–7	21 (12/ 9)	360–440
Belgian Shepherd–Laekenois	20–30	3	6–8	28 (16/ 12)	240–410
Basset Hound	20–32.5	2	5–6	11 (5/ 6)	350–450
Golden Retriever		4	5–10	32 (13/ 19)	360–540
Rhodesian Ridgeback	32–36.5	1	10	10 (7/ 3)	360–550
Old English Sheepdog	30–50	12	2–10	75 (33/ 42)	265–500
Great Dane	50–100	4	3–6	19 (11/ 8)	500–930
Neapolitan Mastiff	50–70	2	9	9 (4/ 5)	600–1,000
All Breeds				345 (175/ 170)	70–1,000

The data from each puppy were first plotted individually and the periods during which the weight showed a steady, smooth increase were visually identified. The values of *P*_0_ needed to generate the curves that better fitted those data were then adjusted by a least squared error approach, using the Solver add‐in of Microsoft Excel.

The performance of the model was evaluated by the following statistical parameters and approaches:

- mean absolute relative error, $\text{MARE}=\frac{\sum \left|\left(O‐P\right)/P\right|}{n}$, where *n* is the number of observations, *O* is the observed weight value and *P* the predicted value by the model
- root mean squared error, $\text{RMSE}=\sqrt{\frac{\sum {\left(O‐P\right)}^{2}}{n}}$
- linear regression forced through the origin, *y = a x*, with *y* being the predicted weight values and *x* the observed ones.

### 2.3 Ethical approval

Ethical approval was not required as all data on daily weight of the puppies were made available by the respective breeders, all hobby breeders, who had collected them for personal use and not specifically for this study.

## 3 RESULTS

The outputs relative to the adjustment of the regression lines are presented in Table 2. The second‐order polynomial was retained as it had the highest *R* ^2^/ adjusted *R^2^* and all the regression coefficients were significantly different from zero. The final model for the weight of a puppy during the first three weeks in the non‐logarithmized form can then be presented as:

(1) $$P=P_{0}\exp\left(0.13084x‐0.001616x^{2}\right)$$

with *P* being the weight (g) on Day *x*, and *P*_0_ the weight (g) on Day zero (birth day).

**Table 2:** Results of the adjustment of the model ln *P* = Σ*a_i_ I_i_* + *b_1_ x* + *b_2_ x* ^2^ to the observed values

Regression statistics	
*R* ^2^	0.9948
Adjusted *R* ^2^	0.9867
Standard error	0.048

Variable	Coefficients	SE	*t* stat	Confidence interval (at 95%)
*x*	0.13084	0.0021	62.018***	0.12667–0.13501
*x* ^2^	−0.001616	7.15 × 10^–5^	22.597***	−0.001758 – −0.001475

**Figure 3:**
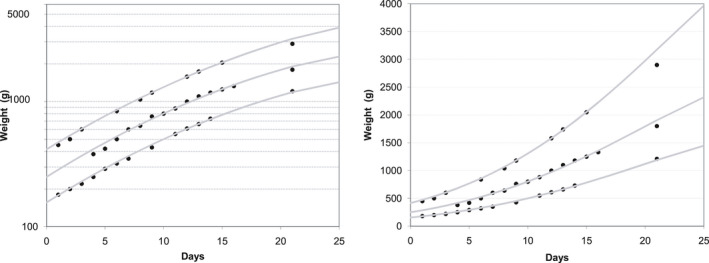
Measured weight values and adjusted curves (Equation 1) for 3 puppies used for model development. Left: in logarithmic scale; right: normal scale. Puppy from litter C (♂): *P*_0_ = 415 g; Puppy from litter A (♂): *P*_0_ = 250 g; Puppy from litter B (♀): *P*_0_ = 156 g

The residuals showed a random distribution around zero for both males and females, confirming that the model is independent of sex. Also, when analysing the residuals for a given puppy, no autocorrelation was detected. Figure 3 shows the measured data and the adjusted curves generated with this model for three different puppies used to develop the model.

Being able to describe puppy growth by a mathematical model is useful as additional information can be easily retrieved such as:

‐ daily weight increase: mathematically, it corresponds to the first derivative of the weight function, and can be calculated as follows:

(2) $$dP/dx=P\left(0.13084‐0.003232x\right)$$

Daily weight gain is thus a function of the weight of the puppy (*P*) on that day, which means that bigger puppies will add more weight than smaller puppies, thus the divergence observed in the respective curves;

‐ relative daily weight increase: can be calculated as follows:

(3) $$\frac{dP/dx}{P}==0.13084‐0.003232x$$

which shows that it is independent of the weight of the puppy and dependent only on its age, decreasing linearly during the first 3 weeks, from 13.1% (on Day 0) to 6.3% (on Day 21);

‐ average relative daily growth rate in the first 21 days:

(4) $$\bar{\left(\frac{dP/dx}{P}\right)}=\frac{\int_{0}^{21}\left(0.13084‐0.003232x\right)dx}{21}=0.097\approx 10\%$$

‐ time to double birth weight:

$$P=2P_{0}\Rightarrow \exp\left(0.13084x‐0.001616x^{2}\right)=2\Leftrightarrow x=5.7\approx 6\;\text{days}.$$

**Figure 4:**
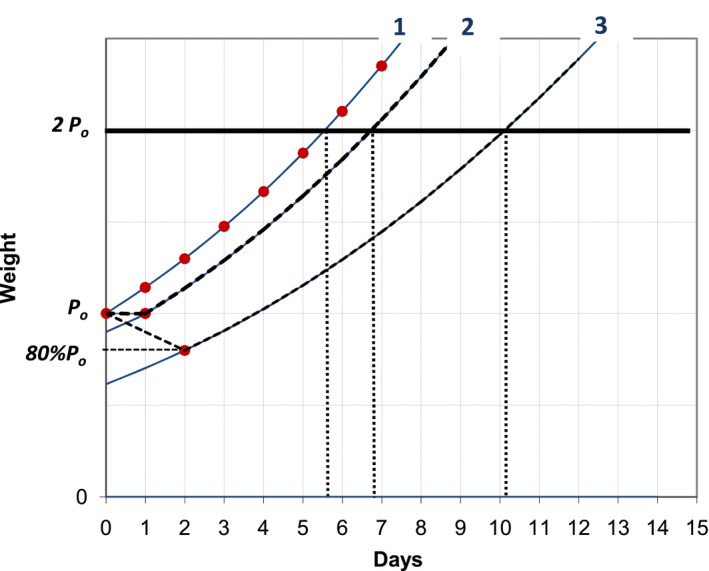
Time to double birth weight as predicted by the model. 1–puppy gaining weight since birth; 2‐ no weight gain during the first 24 hr; 3–loss of 20% of birth weight during the first 48 hr. All the curves are generated by the model given the correspondent *P*_0_ values (*P*_01_ = *P*_0_; *P*_02_ = 0.9 *P*_0_; *P*_03_ = 0.615 *P*_0_)

The model can also predict the time needed to double birth weight if the puppy starts following one of the curves after a stagnation or weight loss during the first days (Figure 4).

**Figure 5:**
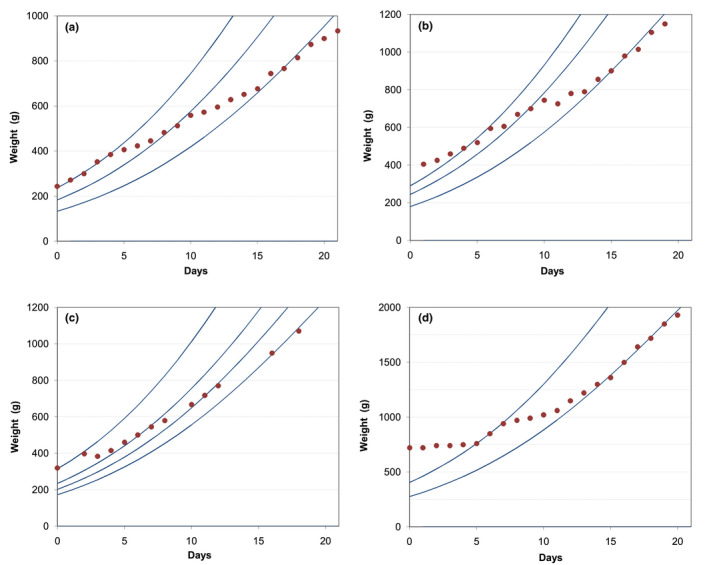
Growth patterns of selected puppies from different breeds. The dots (●) are the weights as measured by the breeders, the lines (─) are generated with Equation (1) giving a value to *P*_0_. (a) Dachshund; (b) Basset Hound; (c) Whippet; (d) Great Dane

**Figure 6:**
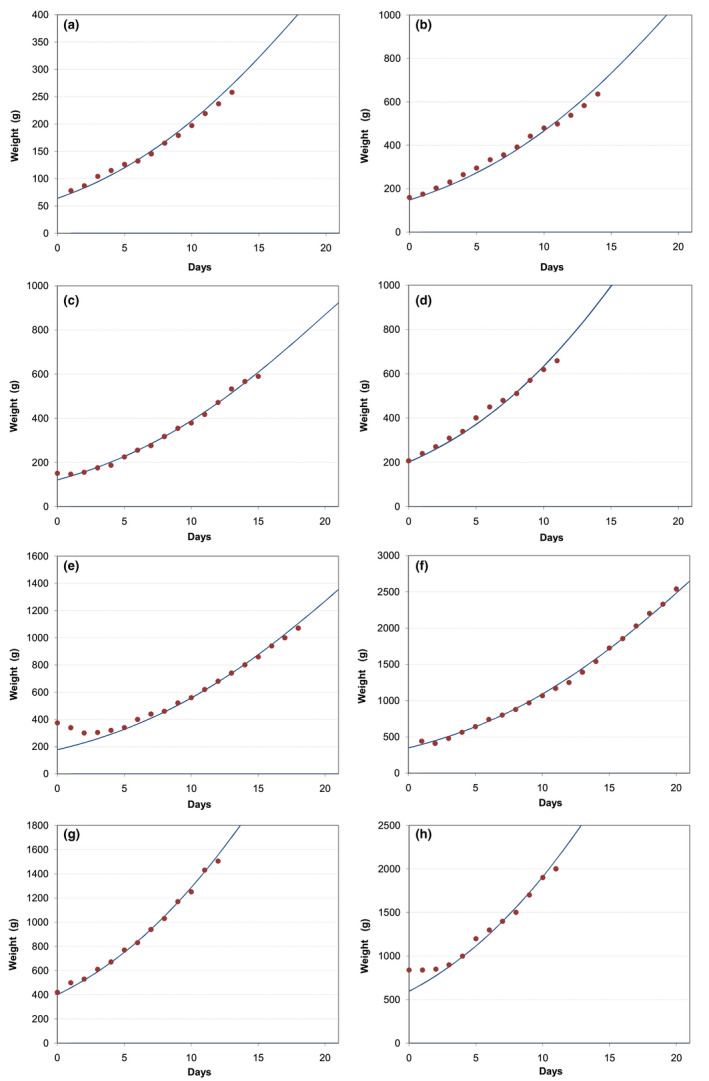
Growth curves of selected puppies from different breeds. The dots (●) are the weights as measured by the breeders, the lines (─) are generated with Equation (1) giving a value to *P*_0_. (a) Yorkshire Terrier (*P*_0_ = 64 g); (b) Portuguese Podengo (*P*_0_ = 148 g); (c) Cavalier King Charles Spaniel (*P*_0_ = 121 g); (d) Shetland Sheepdog (*P*_0_ = 201 g); (e) Bulldog (*P*_0_ = 177 g); (f) Bull Terrier (*P*_0_ = 346 g); (g) Rhodesian Ridgeback (*P*_0_ = 403 g); (h) Great Dane (*P*_0_ = 600 g)

When validating the curves, the puppies from litter D from the OES kennel used to derive the model showed for the first time a different pattern not seen in the previous three litters: during the first 12 days the puppies followed the expected curve but then “fell” to another, lower curve (corresponding to a lower *P*_0_). This drop coincided with the ending of the presential surveillance of the bitch during the night, which probably caused her to leave the nest for longer periods of time, hindering the nursing of the puppies and resulting in a lower than optimum weight increase. It was inferred that any “drop” from the line(s) would therefore indicate something abnormal, or simply not optimal, such as insufficient nursing time, insufficient milk production or low milk quality. It was also observed that, once dropping to a lower line (corresponding to smaller daily absolute growth increments), no puppy returned to the original curve nor any other line corresponding to a higher *P*_0_. This type of weight evolution, with puppies “falling” to lower curves, correspondent to lower *P*_0_, but never returning to the original curve or “jumping” to a higher curve, with higher *P*_0_, was also seen across all breeds (Figure 5), even if supplemented after an initial weight loss (Figure 6e). In all breeds, the number, magnitude and timing of these “falls” did not show any pattern, occurring randomly during the study period.

About 10% of the puppies followed a single line (Figure 6a,b,d,g), as the puppies used for the adjustment of the model, but two thirds of the puppies followed 3 or more lines, dropping from line to line successively (Figure 5b,c) or taking some days until they were able to finally keep on one of the lower curves (Figure 5a,d).

Approximately half of the puppies (48%) gained weight right from Day 0 and started following the line corresponding to their birth weight (Figure 5a,c and Figure 6b,d,g), while about one third of the puppies did not gain weight or lost up to 20% of birth weight during the first 24/48 hr. Almost all puppies (>95%) were able to start following one of the curves up to three days after an initial weight drop (Figure 6c,e,f,h).

**Figure 7:**
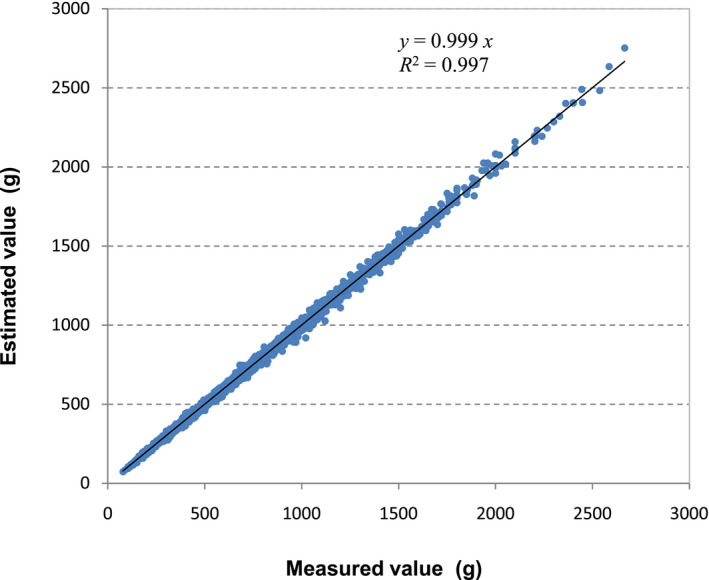
Relationship between measured weights and correspondent estimated values using Equation (1)

In all breeds no difference in growth patterns was detected between males and females, so all data were combined when analysing the performance of the model. Average MARE for all breeds was 2.07%, with similar values across breeds, while RMSE ranged from 4.87 to 36.26 g depending on size (Table 3). Also, both the slope of the adjusted regression line between measured and modelled values and the determination coefficient (*R* ^2^) were very close to 1.0 (Figure 7).

**Table 3:** Goodness of fit of the model assessed by the Mean Absolute Relative Error (*MARE*) (± standard error) and Root Mean Squared Error (*RMSE*) by breed and all breeds combined

Breed	*MARE,* %	*RMSE,* g
Chihuahua	1.52 ± 1.21	4.87
Yorkshire Terrier	2.08 ± 2.03	5.89
Portuguese Podengo	1.93 ± 1.66	9.04
Lhasa Apso	2.30 ± 1.96	13.74
Parson Russell Terrier	1.73 ± 1.35	8.45
Cavalier King Charles Spaniel	2.65 ± 2.32	11.04
Shetland Sheepdog	2.36 ± 1.83	14.05
Standard Dachshund	2.18 ± 1.78	13.96
Whippet	2.04 ± 1.56	13.57
Bulldog	2.25 ± 1.93	17.89
Bull Terrier	1.62 ± 1.60	20.23
Shar Pei	2.20 ± 1.70	19.00
Belgian Shepherd–Laekenois	1.61 ± 0.99	8.63
Basset Hound	2.18 ± 1.71	22.12
Golden Retriever	2.14 ± 1.74	20.54
Rhodesian Ridgeback	2.07 ± 1.62	20.83
Old English Sheepdog	1.98 ± 1.61	22.74
Great Dane	1.79 ± 1.30	22.70
Neapolitan Mastiff	2.69 ± 2.53	36.26
All breeds	2.07 ± 1.54	17.84

## 4 DISCUSSION

When analysing all the data that we had access to, it became evident that only a small percentage of puppies followed a single curve throughout all the period considered (0 to 21 days). This may be one of the reasons why up to now developing growth curves, even for a specific breed, has been elusive. The puppies from the first three litters actually constituted the most appropriate sample to derive the growth curves as they were able to maintain maximum growth rate throughout all the initial 3 to 4 weeks. One of the reasons is surely the fact that the puppies of the first two litters were relatively small, as they belonged to a bigger litter at the start (≥10 puppies), and that there were just two puppies on the third litter. Nursing puppies have an estimated energy requirement that is a linear function of their weight ( (National Research Council [NRC] 2006). Therefore, litters with a smaller total weight, having a smaller total energy requirement, put a lower demand on the lactating bitch that can then provide adequate nutrition to the puppies with a more modest production of milk. In fact, Boutigny et al. (2016) found that growth rates of large breed puppies from large litters can be affected by insufficient milk production/intake.

Since no puppy showed a sustained growth rate higher than the one defined by these curves (that would allow “jumping” to a higher curve, corresponding to a higher *P*_0_) it can be concluded that the model describes maximum growth, irrespective of breed and/or size of the puppy, with maximum relative daily weight gain being properly defined by Equation (3). Such a limit on growth rate means that any reduction in weight gain is not recoverable during this period, and the puppy will inevitably have a smaller weight at the end of this period than the one expected from the initial growth curve (Figure 5). It also explains why smaller puppies at birth do not exhibit a compensatory, higher, growth rate, but rather the same growth rate as their bigger littermates, as observed by Mila et al. (2015) and Tesi et al. (2020).

A guideline often found is that puppies should double their birth weight by one week (Evans & White, 1997), which agrees with the model (Figure 4) and was seen in puppies following a single curve since birth (Figure 6b,d,g). Some other authors (Peterson & Kutzler, 2011; Root Kustriz, 2019), that allow for some weight loss after birth, set this point at 7 to 10 days, which is what the model predicts if the puppy does not gain weight or looses up to 20% of its birth weight during the first 48 hr (Figure 4).

Some studies have reported different growth rates for males and females, with males taking longer to reach adult body weight than females (Allard, Douglass, & Kerr, 1988; Helmink, Shanks, & Leighton, 2000). Though male puppies are heavier than females at 6 weeks old and this difference is maintained until adulthood (Booles, Poore, Legrand‐Defretin, & Burger, 1994; Salt et al., 2017), we did not observe any sexual dimorphism within the first three weeks after birth. Consistently with our results, no differences were evidenced between growth curves of males and females neither in the first 2 days (Mila et al., 2015) nor in the first 6 days after birth (Tesi et al., 2020).

Breed‐specific growth patterns might be expected due to huge variations in size and conformation. Even within the same breed large variations in birth weight exist due to the existence of different lines/types but mostly because of the considerable variation in litter size as affected by age and parity of the bitch, time of mating, number of matings, semen quality, or inbreeding levels (Borge, Tonnessen, Nodtvedt, & Indrebo, 2011; Leroy, Phocas, Hedan, Verrier, & Rognon, 2015) which is negatively correlated with birth weight. Total weight of the litter is in average about 13.5% of the bitch weight after parturition (Meyer, Kienzle, & Dammers, 1985) irrespective of litter size, so the smaller the litter, the larger the individual pups can be (Mila et al., 2015). Even puppies in the same litter can have dissimilar birth weight, as was the case in this study, with large litters having a greater number of low birth weight puppies (Mila et al., 2015).

Although the fact that the curves originally derived for a breed could also be adjusted successfully for other breeds, regardless of weight or conformation, was unexpected, the anterior studies on puppy growth actually pointed in that direction. Effectively, most references consider that puppies, regardless of breed, should gain 5 to 10% of their weight daily (Hoskins, 2001; Kirk, 2001) or even 10 to 15% (Sheffy, 1978), which is in accordance with the model here proposed (Equations 3 and 4). The reason for maximum growth rate being independent of breed may lie in two aspects:

‐ bitch milk composition, although varying throughout the lactation period (Adkins, Lepine, & Lonnerdal, 2001; Oftedal, 1984), does not seem to be breed specific nor related to breed size (Scantlebury, Butterwick, & Speakman, 2000);
‐ the body composition of puppies does not appear to be related to breed size (Meyer, Dammers, & Kienzle, 1985), with the average percentages of dry matter, protein and fat at birth being similar and evolving in the same way with time regardless of breed size (Kienzle, 1998).

**Figure 8:**
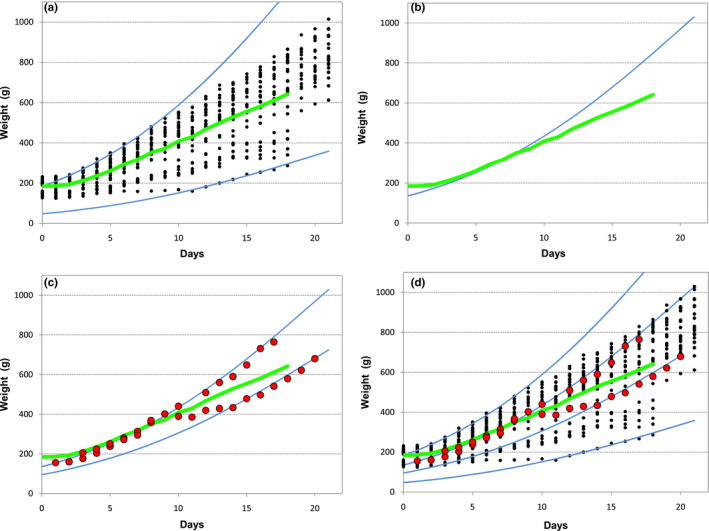
(a) All weight values of all the Cavalier King Charles Spaniel puppies, together with the curve corresponding to the average weight and the 2 limiting curves generated by the model that envelop all the data points (*P*_0_ = 183 g and *P*_0_ = 47 g); (b) Curve representing the average weight and curve generated by the model for *P*_0_ = 135 g; (c) Weight gain of two puppies from two different litters (the two adjusted curves are generated by the model with *P*_0_ = 135 g and *P*_0_ = 95 g). (d) All graphs superimposed. ● measured weight values; 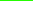 curve representing the average weight; 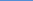 curve generated by the model for a given *P*_0_

Focusing on the growth of individual puppies instead of the whole population is the strength and originality of this study rather than being its weakness. With the huge variation seen in birth weight, even within a same breed, together with the random timing, magnitude of “falling” and number of days needed to resume maximum growth rate, a descriptive statistic, like the average or percentiles, will just reflect and be valid for that sample. Also, the average may represent no actual pattern of growth at all and it will then not constitute a suitable standard for comparison. An example is shown in Figure 8, where it can be seen that the average is influenced by the lack of points representing the higher weight values that would be expected during the last week (Figure 8a). As a consequence, and though the average and the model can give similar results during the first week, they diverge progressively during the second and third weeks (Figure 8b). Figure 8c, depicting the actual growth pattern of two puppies from different litters, is presented to show the finer detail needed for evaluating individual growth that the average or a percentile cannot provide. One of the puppies exhibits maximum growth rate throughout the whole period, while the other puppy “fell” around day 10 and resumed maximum growth rate by day 14. Neither puppy would by then be classified as having an “average” growth, with one being above and the other under average; notwithstanding, both are following the curves corresponding to their actual weight and therefore do not raise any concern nor require any action from the breeder.

Although there is a concern regarding maximum growth rate in puppies from large and giant breeds during the period after weaning (from 2 months to adult age) as it can lead to orthopaedic problems (Hedhammar et al., 1974; Kasstrom, 1975; Kealy et al., 1992; Richardson et al., 2010), it is doubtful that the same applies to the suckling phase. In fact, maximum growth rate during the whole 3 weeks was observed in puppies that were exclusively maternally fed (Figure 3). Contrary to available commercial dog diets, that can be highly variable in nutrient and energy density, bitch milk composition varies within narrow limits (Adkins et al., 2001; Heinze, Freeman, Martin, Power, & Fascetti, 2014; Meyer, Kienzle, et al., 1985; Oftedal, 1984) and overfeeding seems unlikely. Also, while an increased growth rate after weaning is accompanied by overweight, the puppies in this study that maintained maximum growth rate during the first 3 weeks, following only one of the curves (Figures 3 and 6), had similar body condition to puppies that crossed curves, with the heavier puppies simply being bigger in size. These differences in size tend to disappear by weaning time, as verified by Boutigny et al. (2016). Further studies are needed to determine the effects of different growth rates and to define what constitutes optimal growth rate during this phase, but, in the absence of additional data, there seems to be no reason why following one single curve during the first 3 weeks, being a reflection of a total absence of limiting factors, namely milk production by the bitch, should not be sought after.

**Figure 9:**
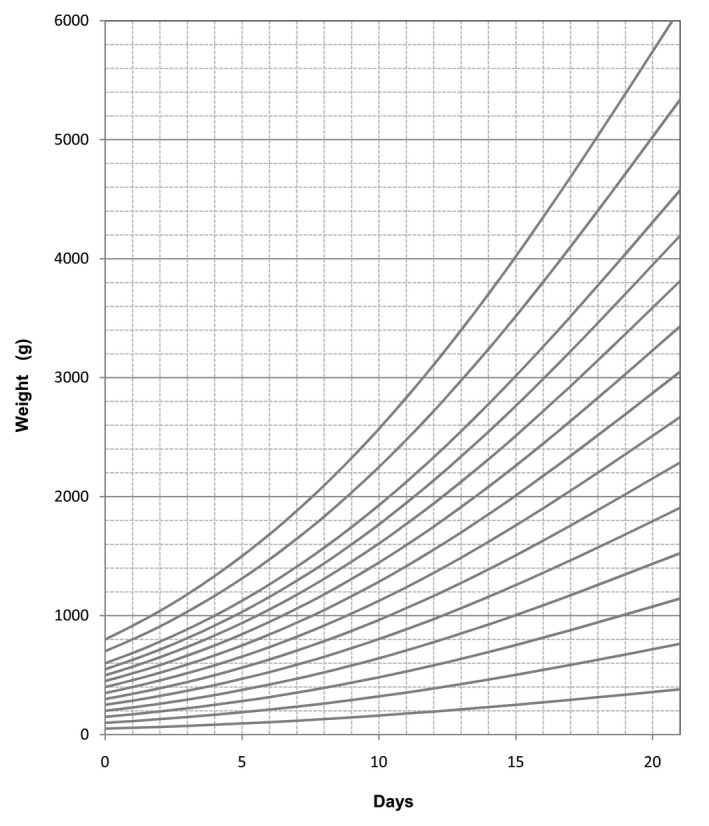
Growth curves according to weight at Day 0 (Equation 1)

Equation (1) is more flexible to use than a growth chart but it can also be easily converted to that form (Figure 9) if that is the preference of the user.

The fact that the model proposed was validated by data gathered by the breeders themselves, and reflecting different management conditions of puppies and lactating bitches (housing, environment or feeding), supports its adequacy for use by breeders or veterinarians in monitoring puppy growth in actual field conditions.

## CONFLICT OF INTEREST

The author has no conflict of interests to declare.

## AUTHOR CONTRIBUTION

Isabel Alves: Conceptualization; Data curation; Formal analysis; Writing‐original draft; Writing‐review & editing.

## ETHICAL STATEMENT

Ethical approval was not required as all data on daily weight of the puppies were made available by the respective breeders, all hobby breeders, who had collected them for personal use and not specifically for this study.

## Data Availability

The data that support the findings of this study are available on request from the corresponding author. The data are not publicly available due to privacy restrictions.
